# Intestinal Nonrotation and Cecal Volvulus: A Unique Combination of Rare Pathologies

**DOI:** 10.7759/cureus.39761

**Published:** 2023-05-31

**Authors:** Tatiana Fernandez Trokhimtchouk, Luis F Flores, Álvaro Morillo Cox, Alberto Gordillo, Joseline K Crespo Martinez

**Affiliations:** 1 General Surgery, Universidad Internacional del Ecuador/Axxis Hospital, Quito, ECU; 2 General Surgery, Axxis Hospital, Quito, ECU

**Keywords:** laparotomy, volvulus of midgut, hemicolectomy, intestinal obstruction, intestinal volvulus

## Abstract

Intestinal nonrotation and cecal volvulus are rare clinical entities, and their coexistence is exceptionally uncommon. We present a case of a 41-year-old male patient with symptomatic intestinal nonrotation and associated cecal volvulus. Diagnostic imaging played a crucial role in recognizing the conditions and guiding surgical intervention. The patient underwent laparotomy and right hemicolectomy with a favorable postoperative course. This case highlights the challenges in diagnosing and managing these rare conditions. Further studies are needed to optimize the management strategies for this unique combination of pathologies.

## Introduction

Cecal volvulus is a rare cause of large bowel obstruction that results from an axial twisting of the redundant cecum around its mesentery or its upward folding resulting in a closed loop. Over time, it may progress to strangulation, ischemia, and sepsis, with potentially deleterious consequences. It accounts for 30% of all colonic volvulus, occupying second place after sigmoid one, and has a peak incidence in the elderly [1].

Normal development of the embryonic gut includes a 270° counterclockwise rotation that occurs in three phases, around the superior mesenteric pedicle [2]. When this process fails, the condition is called malrotation. The prevalence of malrotation is estimated to be around one in 500 live births, with a higher incidence in Caucasians and a male predominance [2,3]. It can be classified into different subtypes, but for practical purposes, it is often simplified into nonrotation and malrotation [4]. Nonrotation occurs when the midgut returns to the peritoneal cavity without undergoing normal rotation, resulting in the small intestine being positioned on the right side of the abdomen and the colon on the left side. Most cases of malrotation are present during the first years of life, while it occurs in 0.2%-0.5% of the adult population. Although this condition can remain asymptomatic and be discovered incidentally, it can also lead to volvulus, typically occurring at the duodenojejunal junction (DJJ) or mid transverse colon [1].

The coexistence of nonrotation and cecal volvulus is exceedingly rare. Diagnostic imaging plays a critical role in identifying intestinal nonrotation and its complications. Plain abdominal radiographs and computed tomography (CT) are commonly used for evaluation. Findings that may suggest intestinal nonrotation include a left-sided cecum, reversed relationship between the superior mesenteric artery (SMA) and superior mesenteric vein (SMV), and malposition of the DDJ [1,3,4]. While incidental findings of intestinal nonrotation do not require treatment in adults, surgical resection is the definitive management for cecal volvulus [5].

We report the case of a 41-year-old male patient who presented with symptoms suggestive of intestinal obstruction, and was found to have an intestinal nonrotation with associated cecal volvulus on imaging. He was managed by laparotomy and right hemicolectomy, after which had an uneventful postoperative recovery.

## Case presentation

**Figure 1:**
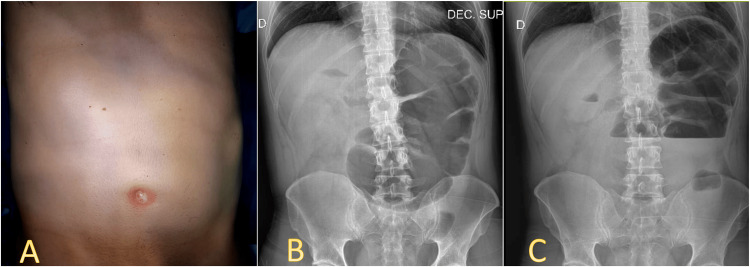
(A) Abdominal distention on physical examination. A plain radiograph (B) on the decubitus position, with a markedly dilated cecum located in the left hemiabdomen, (C) In the erect position with fluid levels present.

A 41-year-old male patient with no significant medical history presented to the emergency room with a complaint of colicky abdominal pain lasting for 24 hours. The pain was diffuse and mild, accompanied by nausea, and he experienced one episode of vomiting. The patient noted the absence of flatus and bowel movements since the onset of pain. He attempted self-medication with oral hyoscine butylbromide but found no relief. Vital signs were within normal limits. On abdominal examination, mild distension was observed (Figure 1A), bowel sounds were absent, percussion yielded a tympanic sound, and diffuse pain was elicited upon palpation without signs of peritonism. A digital rectal examination revealed an empty rectal ampulla. Laboratory analyses showed neutrophilia (87.7%) with no leukocytosis (10,580/mm^3^), lactate dehydrogenase of 159 (normal range 102-341 IU/L), normal lactic acid of 1.2 mmol/L, serum electrolytes within range, and no other relevant findings. Based on the clinical presentation, a preliminary diagnosis of intestinal obstruction was made. A plain abdominal radiograph, shown in Figures 1B, 1C, was requested and revealed a markedly dilated cecum displaced to the left flank and hypochondrium.

**Figure 2:**
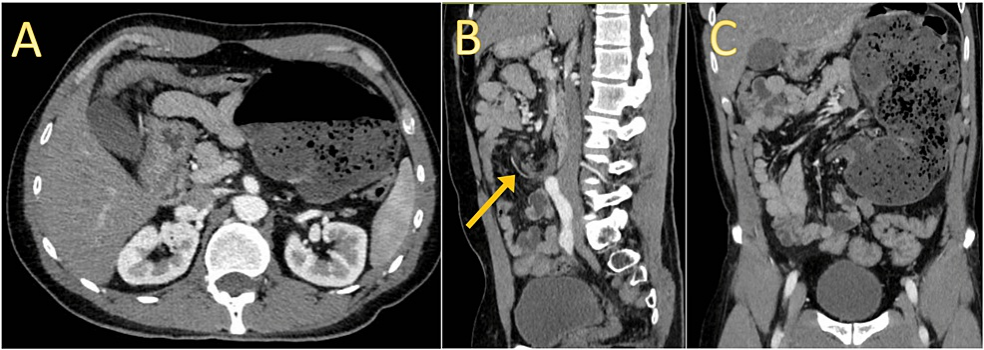
Abdominal contrast-enhanced CT. (A) Axial view in the arterial phase, the cecum is dilated (measured at 10.6 cm) in the left hypochondrium. (B) Sagittal view where the twisting of the mesentery is visible (yellow arrow). (C) Coronal view with congestion of the mesentery and fat stranding.

**Figure 3:**
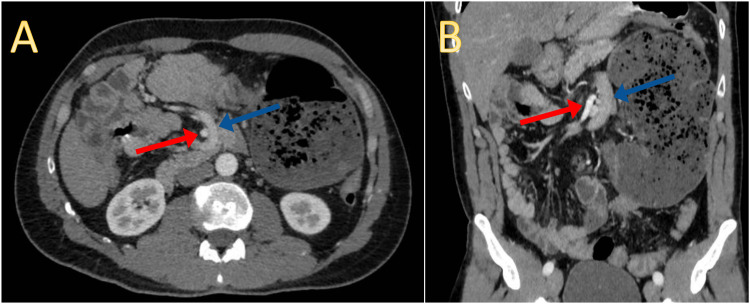
Abdominal contrast enhanced CT in axial (A) and coronal (B) views showing reversed relationship of the SMA (red arrow) and SMV (blue arrow), a pathognomonic sign of intestinal nonrotation.

To further evaluate the condition, an abdominal contrast-enhanced CT scan was performed, which indicated signs suggestive of cecal volvulus and intestinal nonrotation (Figures 2A-2C, 3A, 3B). No evidence of perforation, bowel wall edema, or free fluid was observed.

**Figure 4:**
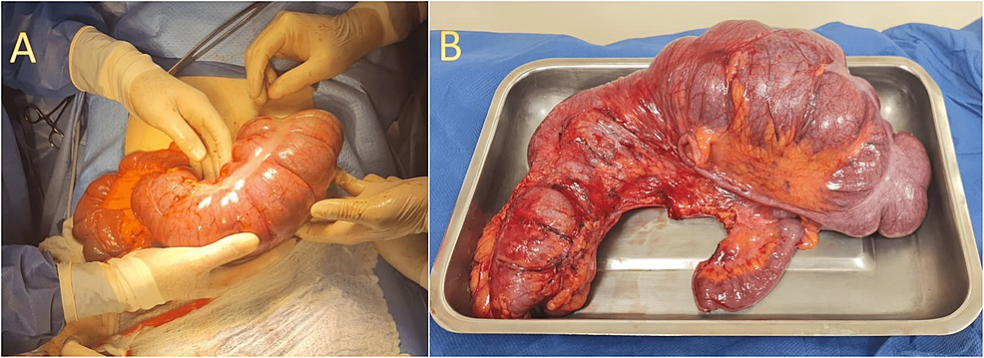
(A) Transoperative photograph of the volvulated cecum. (B) Resected surgical specimen.

The patient was taken to the operating room for an emergency laparotomy. A midline incision was made, and surgical findings included a markedly dilated cecum upwardly folded to the left hypochondrium with a 360° clockwise twisting upon its mesentery; no signs of necrosis or free liquid were noted; and no Ladd bands were found (Figures 4A, 4B). A right hemicolectomy was performed with a primary ileotransverse latero-lateral anastomosis.

The postoperative period was uneventful, with no complications observed. The patient was discharged home on the third postoperative day, resuming his normal diet and experiencing regular bowel movements.

## Discussion

Intestinal nonrotation is a rare congenital condition that results from abnormal embryological development of the gut and is mostly found in children. When found in adults incidentally, without associated symptoms, treatment is unnecessary. However, there have been a few reported cases of symptomatic nonrotation that require specific surgical management [6].

Cecal volvulus is an uncommon condition that can lead to substantial morbidity if not promptly diagnosed and treated. It is associated with cecal hypermobility, which occurs due to the failure of fusion between the parietal and visceral peritoneum of the right colon. This results in a loose mesentery that allows the cecum to fold upward and twist axially upon itself, leading to cecal bascule and axial cecal volvulus, respectively [1,2]. 

Regarding the coexistence of these two pathologies, it appears to be a highly unique combination. We were able to find only one case report by Pramod et al., describing a 15-year-old male with acute abdominal pain diagnosed of cecal volvulus and associated malrotation. The patient underwent a Ladd's procedure for management [5].

In the case we present, cecal volvulus was associated with nonrotation, the latter occurs when the physiological 270° counterclockwise rotation of the gut fails. As a result, the small intestine and the DJJ remain on the right side of the midline, while the large bowel remains on the left [1].

Diagnostic imaging, particularly abdominal radiographs and CT scans, plays a crucial role in evaluating symptomatic nonrotation. Plain abdominal radiographs may reveal nonspecific findings unless complications such as volvulus are present. In our case, the marked dilation of the cecum was clearly visible in the left hemiabdomen, along with signs of large bowel obstruction, as shown in Figure 1. 

CT scans provide more detailed information, allowing for identification of the characteristic signs: abnormal position of the DJJ and inversion of the SMA and SMV, the latter being at the left [1,3,4,5,7]. These imaging modalities aid in confirming the diagnosis of complicated intestinal nonrotation and help determine the need for surgical intervention.

Surgical management is the primary treatment approach for cecal volvulus, typically involving resection of the twisted bowel segment [4]. Conversely, the optimal management strategy for nonrotation without volvulus remains debatable due to its rarity and the lack of consensus. In cases of asymptomatic nonrotation, incidental discovery during unrelated surgical procedures or imaging studies may not necessitate immediate intervention. However, in symptomatic cases or when complications such as volvulus arise, surgical intervention is necessary to alleviate the obstruction and prevent further morbidity and mortality [6,8].

In the reported case, the patient underwent a laparotomy and right hemicolectomy for resection of the volvulated cecum with the creation of a primary anastomosis. The successful surgical intervention resulted in the resolution of symptoms and a favorable postoperative course with no complications.

Given the rarity of intestinal nonrotation and cecal volvulus, it is crucial to raise awareness among healthcare providers to facilitate early diagnosis and management. Timely intervention can significantly reduce the risk of complications such as bowel ischemia, perforation, and sepsis, which carry substantial morbidity and mortality.

## Conclusions

In conclusion, we report a rare case of a 41-year-old male patient with symptomatic intestinal nonrotation and associated cecal volvulus. This combination of pathologies is exceedingly uncommon, posing diagnostic and management challenges. Abdominal contrast-enhanced CT scan played a crucial role in recognizing the conditions and guiding the conduct. Surgical management, through laparotomy and right hemicolectomy, successfully resolved the symptoms and led to an uneventful postoperative course. Further studies are needed to enhance our understanding of optimal management strategies for these unique clinical entities.
